# Comparison of methods for the analysis of therapeutic immunoglobulin G Fc-glycosylation profiles—Part 2: Mass spectrometric methods

**DOI:** 10.1080/19420862.2015.1045173

**Published:** 2015-05-21

**Authors:** Dietmar Reusch, Markus Haberger, David Falck, Britta Peter, Bernd Maier, Jana Gassner, Michaela Hook, Katharina Wagner, Lea Bonnington, Patrick Bulau, Manfred Wuhrer

**Affiliations:** 1 Pharma Biotech Development Penzberg; Roche Diagnostics GmbH; Penzberg, Germany; 2 Center for Proteomics and Metabolomics; Leiden University Medical Center; Leiden, The Netherlands; 3 Division of BioAnalytical Chemistry; Department of Chemistry and Pharmaceutical Sciences; VU University Amsterdam; Amsterdam, The Netherlands

**Keywords:** IgG glycosylation, monoclonal antibody (mAb), HILIC-UPLC, method comparison, mass spectrometry, ESI-MS, MALDI-MS, LC-MS, glycan analysis

## Abstract

To monitor the Fc glycosylation of therapeutic immunoglobulin G in bioprocess development, product characterization and release analytics, reliable techniques for glycosylation analysis are needed. Several analytical methods are suitable for this application. We recently presented results comparing detection methods for glycan analysis that are separation-based, but did not include mass spectrometry (MS). In the study reported here, we comprehensively compared MS-based methods for Fc glycosylation profiling of an IgG biopharmaceutical. A therapeutic antibody reference material was analyzed 6-fold on 2 different days, and the methods investigated were compared with respect to precision, accuracy, throughput and analysis time. Emphasis was put on the detection and quantitation of sialic acid-containing glycans. Eleven MS methods were compared to hydrophilic interaction liquid chromatography of 2-aminobenzamide labeled glycans with fluorescence detection, which served as a reference method and was also used in the first part of the study. The methods compared include electrospray MS of the heavy chain and Fc part after limited digestion, liquid chromatography MS of a tryptic digest, porous graphitized carbon chromatography MS of released glycans, electrospray MS of glycopeptides, as well as matrix assisted laser desorption ionization MS of glycans and glycopeptides. Most methods showed excellent precision and accuracy. Some differences were observed with regard to the detection and quantitation of low abundant glycan species like the sialylated glycans and the amount of artefacts due to in-source decay.

## Abbreviations

PNGase F: Peptide-*N*-Glycosidase F
mAb: monoclonal antibody
Fc: fragment crystallizable
HILIC-UHPLC: hydrophilic interaction liquid chromatography-ultra high performance liquid chromatography
2-AB: 2-aminobenzamide
Fab: fragment antigen-binding
IgG: immunoglobulin G
PGC-MS: porous graphitized carbon chromatography- mass spectrometry
ESI-MS: electrospray ionization-mass spectrometry
MALDI: matrix assisted laser desorption ionization
IdeS protease: proteolytic enzyme like protease from Streptococcus pyrogenes
HPAEC-PAD: high-performance anion exchange chromatography with pulsed amperometric detection
RP-HPLC: reversed phase high performance liquid chromatography
CE: capillary electrophoresis
TIC: total ion chromatogram
LCMS: liquid chromatography-mass spectrometry

## Introduction

Recombinant monoclonal antibodies (mAbs) are efficacious therapeutic agents for various diseases. MAbs are glycoproteins typically carrying 2 biantennary N-glycans in the Fc part. The Fc glycosylation pattern greatly affects the mAb effector functions.^1-4^ There are many state-of-the-art analytical methods available to monitor IgG Fc-glycosylation. Mass spectrometry (MS) is particularly suited for analyzing complex glycans or glycopeptide mixtures, and it has the intrinsic power to deduce information about the composition of the glycospecies from their molecular mass. All MS-based methods offer advantages and disadvantages for analyzing glycosylation variants, as outlined in recent reviews.^5-9^ When a separation technique is coupled online to electrospray ionization (ESI)-MS, the standardized migration or elution position can also be used for glycospecies assignment. In principle, the MS-based methods for this application can be sub-divided into 3 categories: 1) top-down or middle-down analysis by characterizing the IgG molecule with ESI-MS either after reduction of the disulfide bonds or after digestion with a proteolytic enzyme such as IdeS protease (FabRICATOR®, from *Streptococcus pyogenes*)^10-12^ 2) bottom-up analysis by proteolytic digesting the IgG molecule, e.g., with trypsin, and measuring the glycopeptides either with or without prior separation, using ESI-MS or matrix-assisted laser desorption ionization (MALDI)-MS detection, respectively;^13-15^ and 3) enzymatic release of the Fc-glycans and measurement, with or without prior separation by high performance liquid chromatography (HPLC), followed by respective ESI-MS or MALDI-MS analysis of the glycans.^16-18^

While most of the mass spectrometric analyses of glycans and glycopeptides are performed in positive-ion mode, both negative-mode MALDI-MS and ESI-MS have recently been applied very successfully with improved ionization efficiency, reduced cation adduct formation, and highly informative fragmentation patterns including diagnostic ions valuable for structural analysis.^16,19^

The analysis of sialic acid by MS is challenging because the ionization may be different compared to neutral glycans. Furthermore, sialic acid-containing glycans are even more prone to fragmentation than neutral glycans, which results in in-source or metastable decay. Therefore, sialic acids are often removed prior to MS analysis and measured separately, e.g., by reversed phase-HPLC with fluorescence detection (RP-HPLC-FLD)^20,21^ or high-performance anion exchange chromatography with pulsed amperometric detection (HPAEC-PAD).^22^ Derivatization may be used to neutralize and stabilize sialic acids and enable sialic acid analysis by MS.^23^

We recently published a comparison of non-MS, separation-based methods for glycan analysis of a mAb by enzymatic glycan release followed by HPLC or capillary electrophoresis (CE)-separation after fluorescent labeling, or by HPAEC-PAD.^24^ Here, we describe a thorough comparison of MS-based methods for glycan analysis using the same mAb sample. The study involved 2 laboratories, a biopharmaceutical company (Roche Diagnostics GmbH), and an academic research laboratory (Leiden University Medical Center). The mAb sample was analyzed 6-fold on 2 different days. Eleven MS-based methods were evaluated for the analysis of the Fc glycosylation, with 7 methods using ESI ionization and 4 methods employing MALDI ionization. Two methods detected glycosylated polypeptides after reduction or limited proteolytic cleavage at the IgG hinge region, 6 methods measured tryptic glycopeptides, and 3 methods analyzed PNGase F-released N-glycans. MS-based methods were compared with each other as well as with HILIC-UHPLC profiling of 2-aminobenzamide (2-AB)-labeled glycans employing fluorescence detection, which served as a reference method. Special attention was paid to the measurement of low sialylation levels. An overall conclusion for all methods (non-MS and MS-based) evaluated in the entire study will also be presented here.

## Results

**Figure 1.**
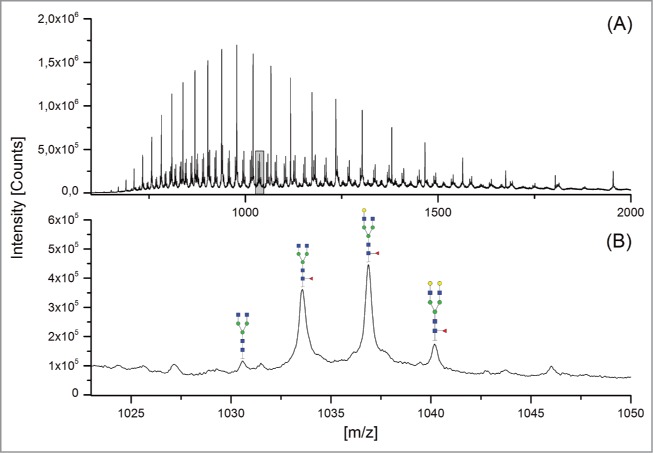
ESI-MS Heavy Chain. (**A**) Overview mass spectrum; (**B**) zoomed spectrum showing the heavy chain ions with z = 49.

**Figure 2.**
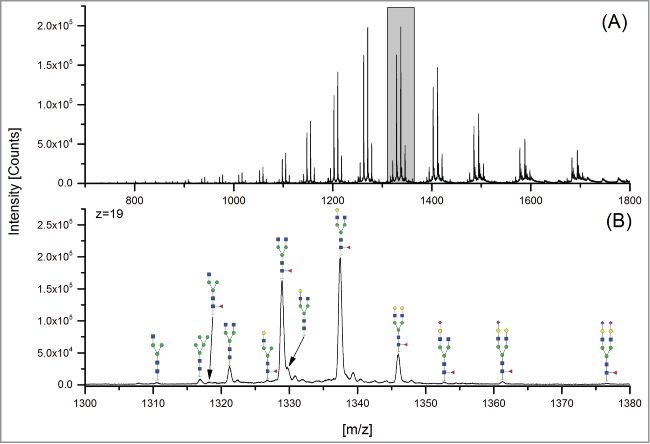
ESI-MS after IdeS (FabRICATOR® IdeS protease). (**A**) Overview mass spectrum; (**B**) zoom showing the species of z = 19.

**Figure 3.**
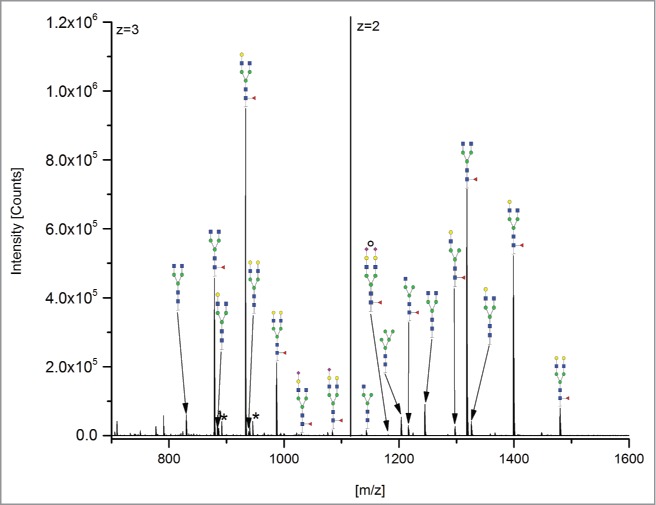
ESI-MS Glycopeptides; * = adducts; ° species with z = 3.

**Figure 4.**
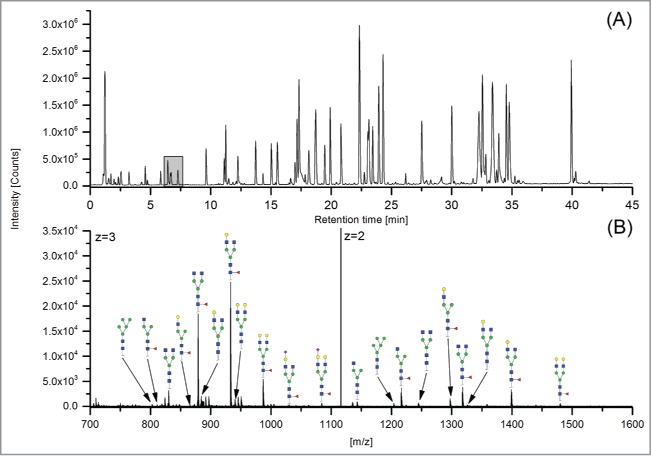
LCMS with Q-TOF. (**A**) TIC; (**B**) MS spectrum of 7.57 – 7.95 min; * = adducts.

**Figure 5.**
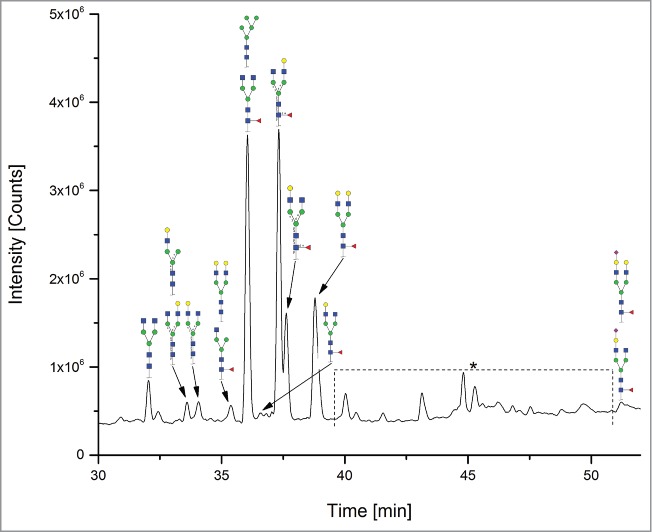
PGC-MS. *= not identified peaks.

**Figure 6.**
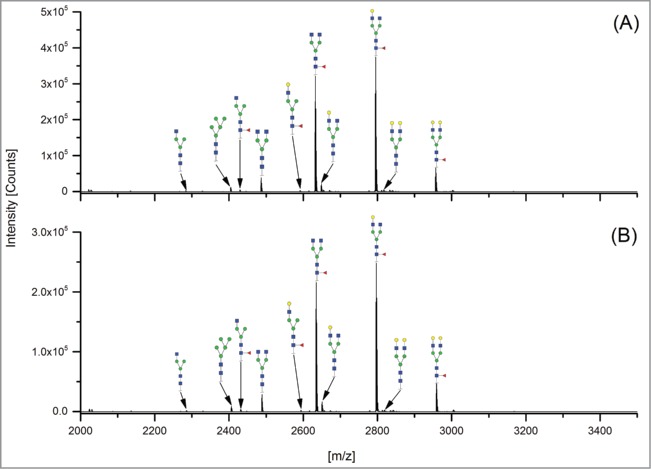
MALDI-MS Glycopeptides performed in (**A**) positive ion mode on N-glycosylated variants of peptide EEQYNSTRY [M+H]^+^ and (**B**) negative ion mode on [M−H]^−^.

**Figure 7.**
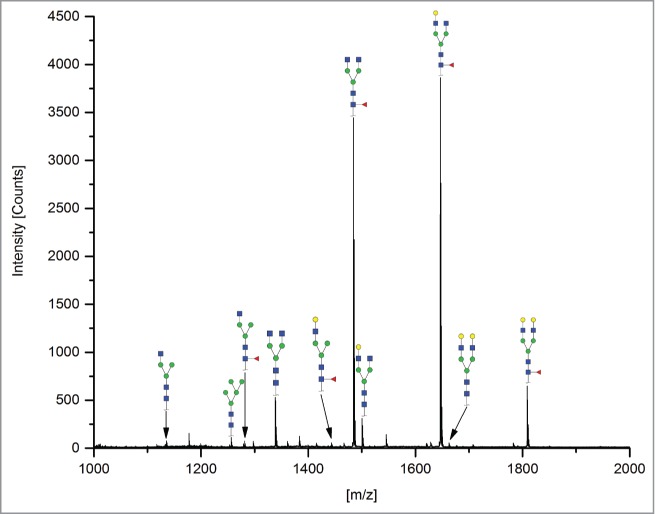
MALDI-MS Glycans. N-glycans were released by PNGase F, and sodium adducts were detected by positive ion mode MALDI-TOF-MS.

**Figure 8.**
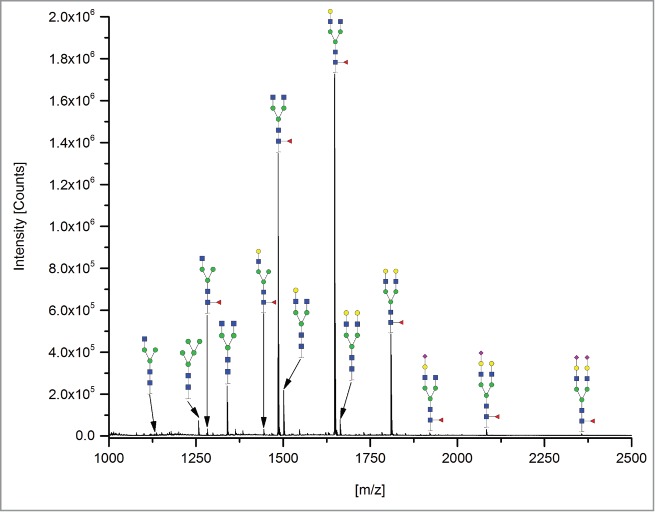
MALDI-MS Stabilized Glycans. N-glycans were released by PNGase F, sialic acids were stabilized by lactonization, and sodium adducts were detected by positive ion mode MALDI-TOF-MS.

The 12 methods used in this study for quantifying Fc glycosylation are listed in **Table 1**: 1) The reference method HILIC(2-AB); 2) ESI-MS Heavy Chain – ESI-MS after reduction of disulfide bonds (**Fig. 1**); 3) ESI-MS after IdeS – ESI-MS following digestion with IdeS protease (**Fig. 2**); 4) ESI-MS Glycopeptides - ESI-MS of purified glycopeptides (**Fig. 3**); 5) LC-MS with Orbitrap - LCMS of glycopeptides in a tryptic digest using an Orbitrap™ mass spectrometer (**Fig. S1**); 6) LCMS with Q-TOF - LCMS of a tryptic digest using a Synapt G2® quadrupole-TOF-MS (**Fig. 4**); 7) Nano-LCMS with Q-TOF – fast nano-LCMS of a tryptic digest using a Maxis impact™ quadrupole-TOF-MS (**Fig. S2**); 8) PGC-MS - Separation with porous graphitized carbon (PGC) chromatography and detection with ESI-MS (**Fig. 5**); 9) Positive MALDI-MS Glycopeptides - MALDI-MS of glycopeptides with detection in the positive ion mode (**Fig. 6A**); 10) Negative MALDI-MS Glycopeptides - MALDI-MS of glycopeptides in negative ion mode (**Fig. 6B**); 11) MALDI-MS Glycans - MALDI-MS in positive ion mode of released glycans (**Fig. 7**); and 12) MALDI-MS Stabilized Glycans - MALDI-MS in positive ion mode of released glycans after stabilization of sialic acid (**Fig. 8**).

**Table 1.** Overview of used methods

Nr.	Method	Lab	Description	Sample[µg]
1	HILIC(2-AB)	1	2-AB labeling of released glycans; separation with HILIC-UPLC (LIF detection)	200
2	ESI-MS Heavy Chain	1	Reduction of mAb with TCEP and direct infusion with ESI-MS	250
3	ESI-MS after IdeS	1	Digestion with IdeS-Protease (Fabricator®) and direct infusion with ESI-MS	100
4	ESI-MS Glycopeptides	1	Tryptic digestion and direct infusion with ESI-MS after purification with Sepharose beads (HILIC)	50
5	LCMS with Orbitrap	1	LCMS after tryptic digestion with an Orbitrap™ mass spectrometer (Thermo Fisher™ Scientific Inc.)	350
6	LCMS with Q-TOF	1	LCMS after tryptic digestion with mass spectrometer Synapt® G2 q-TOF (Waters Corp.)	350
7	Nano-LCMS with Q-TOF	2	LCMS of tryptic digestion with mass spectrometer Maxis impact™ q-TOF, (Bruker Corp.)	20
8	PGC-MS	1	Release of glycans with PNGase F, separation with PGC coupled to ESI-MS	450
9	Positive MALDI-MS Glycopeptides	2	Tryptic digestion, HILIC-SPE and positive ion mode MALDI-TOF-MS	20
10	Negative MALDI-MS Glycopeptides	2	Tryptic digestion, HILIC-SPE and negative ion mode MALDI-TOF-MS	20
11	MALDI-MS Glycans	1	Release of glycans with PNGase F and measurement with MALDI-TOF-MS	75
12	MALDI-MS Stabilized Glycans	2	Release of glycans with PNGase F and measurement with MALDI-TOF-MS after ethyl esterification	50

### Assignment of glycostructures

Peak assignment for the reference method HILIC(2-AB) was previously described.^24^ Peak assignment for the MS-based methods was done on the basis of the molecular composition (in terms of hexoses, N-acetylhexosamines, deoxyhexoses and N-acetylneuraminic acids) and previous structural characterization, including chromatographic and electrophoretic methods in combination with glycan standards.^24^ For the ESI-MS Heavy Chain method, the glycan composition of the heavy chain (HC) was deduced by subtracting the mass expected for a non-glycosylated HC from the molecular masses of the observed glycosylated species. The same holds true for ESI-MS after IdeS. For the glycopeptide analysis (LCMS methods, ESI-MS Glycopeptides and MALDI-MS Glycopeptides), the expected molecular masses of the glycopeptides were taken for peak assignment.

### Detected glycosylation features

With the exception of ESI-MS Heavy Chain, all methods facilitated detection of the main Fc N-glycan species that are typically found on therapeutic IgG mAbs produced in Chinese hamster ovary (CHO) cells (G0F, G1F, G2F, G0, G1 and M5; see **Table 2** for key). This holds also true for the separation-based methods used in the first part of the study. The HILIC(2-AB) reference method allowed the assignment of 15 peaks with differentiation between monogalactosylated species with upper (α1,6) versus lower (α1,3) arm linked galactosylation, as also reported in our previously published study.^24^ In contrast to HILIC(2-AB) as well as other chromatographic separation methods,^24^ the MS-based detection methods intrinsically do not allow the distinction between the monogalactosylated isomers. Of the MS-based methods included in the comparisons made here, only PGC-MS distinguished the 2 monogalactosylated species due to the chromatographic separation involved. In principle the LC-MS analysis of a tryptic digest methods should also resolve monogalactosylated species.^25^ However, we have not optimized the LC-MS separation for resolution of glycosylated peptides. Thus, we were not able to separately quantify the 2 species. For the relative quantitation of glycans with the MS-based methods, the sum of the 2 isomeric monogalactosylated peaks was taken into account, with a total of 14 glycan structures being detected (**Table 2**).

**Table 2.** Quantitative evaluation of method performance. Each analytical method was applied in 2 separate series 6 replicates per batch. Relative abundance of the various glycan species are given in percent, with standard deviations in parentheses. Key: H, hexose; N, N-acetylhexosamine; F, deoxyhexose; S, N-acetylneuraminic acid (sialic acid); G0F-N, agalacosylated, core-fucosylated, monoantennary species, *etc*.; n.d.: not detected; n.a.: not applicable. n,q.: not quantifiable

Glycan species	Structural scheme	HILIC(2-AB)Reference Method	ESI-MS Heavy Chain	ESI-MS after IdeS	ESI-MS Glycopeptides	LCMS with Orbitrap	LCMS with Q-TOF	nano-LCMS with Q-TOF	PGC-MS	Positive MALDI_MS Glycopeptides	Negative MALDI-MS Glycopeptides	MALDI-MS Glycans	MALDI-MS Stabilized Glycans
G0F[H3N4F1]		35.5 (0.1)35.3 (0.1)	34.9 (0.2)34.7 (0.2)	31.3 (0.5)32.0 (0.3)	29.3 (0.2)30.6 (0.7)	29.8 (0.2)32.4 (0.3)	29.7 (0.4)29.9 (0.3)	32.4 (0.5)33.6 (0.9)	36.5 (0.4)36.2 (0.2)	37.8 (0.9)38.2 (0.3)	38.0 (0.9)38.4 (0.1)	37.3 (0.2)37.6 (0.5)	30.6 (0.7)31.2 (0.2)
G1F[H4N4F1]		43.4 (0.1)43.3 (0.1)	45.5 (0.2)45.6 (0.2)	39.5 (0.3)39.6 (0.3)	40.6 (1.0)38.6 (0.4)	38.4 (0.1)39.2 (0.3)	40.1 (0.4)40.1 (0.3)	42.4 (0.3)40.5 (1.8)	42.7 (0.4)42.6 (0.2)	46.6 (0.5)46.5 (0.1)	47.0 (0.5)46.9 (0.2)	44.4 (0.6)44.4 (0.6)	46.1 (0.7)47.9 (0.3)
G2F[H5N4F1]		9.5 (<0.1)9.6 (0.1)	10.7 (0.1)11.1 (0.1)	11.9 (0.5)11.1 (0.3)	9.8 (0.2)8.6 (0.4)	10.1 (0.1)9.3 (0.1)	9.0 (0.1)9.0 (0.1)	9.7 (0.3)9.0 (0.3)	9.2 (0.1)9.2 (0.1)	7.3 (0.5)7.3 (0.2)	7.0 (0.4)7.0 (0.1)	7.0 (0.1)6.6 (0.3)	9.9 (0.5)9.0 (0.3)
G0[H3N4]		4.6 (0.1)4.7 (0.1)	9.0 (0.3)8.5 (0.2)	6.0 (0.1)6.0 (0.1)	5.1 (0.3)5.9 (0.3)	6.1 (<0.1)6.6 (<0 .1)	4.4 (0.1)4.6 (0.1)	4.9 (0.3)5.8 (0.5)	3.6 (0.1)3.8 (0.1)	3.6 (0.2)3.8 (<0.1)	3.4 (0.1)3.7 (0.1)	5.0 (0.1)4.8 (0.2)	4.3 (0.2)4.0 (0.2)
G1[H4N4]		3.3 (<0.1)3.4 (<0 .1)	n.d.n.d.	n.q.n.q.	3.7 (0.2)3.8 (0.1)	4.7 (0.1)4.8 (<0.1)	3.0 (0.1)3.0 (0.2)	3.3 (0.2)3.6 (0.2)	2.5 (<0.1)2.6 (<0.1)	2.1 (0.1)2.0 (0.1)	2.0 (0.1)1.9 (<0.1)	3.0 (0.1)3.0 (0.1)	4.1 (0.1)3.8 (0.1)
G2[H5N4]		0.3 (<0 .1)0.4 (<0.1)	n.d.n.d.	n.d.n.d.	1.1 (0.1)1.0 (0.1)	0.7 (<0 .1)0.6 (0.1)	0.4 (<0 .1)0.4 (<0.1)	0.9 (0.1)0.9 (0.1)	0.5 (<0 .1)0.4 (<0.1)	0.3 (<0 .1)0.2 (<0.1)	0.3 (<0 .1)0.2 (<0.1)	0.5 (0.1)0.5 (0.1)	1.5 (0.1)1.3 (0.2)
G0F-N[H3N3F1]		0.5 (<0.1)0.5 (<0.1)	n.d.n.d.	1.1 (0.1)1.2 (0.1)	2.7 (0.2)3.7 (0.2)	2.5 (0.1)1.2 (<0.1)	5.6 (0.1)5.6 (0.1)	1.0 (<0.1)0.9 (<0.1)	0.5 (<0.1)0.5 (<0.1)	0.4 (<0.1)0.3 (<0.1)	0.4 (<0.1)0.3 (<0.1)	0.6 (0.1)0.8 (0.1)	<0.1 (<0.1)<0.1 (<0.1)
G1F-N[H4N3F1]		n.d.(n.d.)	n.d.n.d.	2.4 (0.1)2.6 (0.1)	2.7 (0.2)2.4 (0.1)	1.3 (<0.1)0.9 (<0.1)	2.7 (0.1)2.6 (0.1)	0.7 (<0.1)0.6 (<0.1)	0.6 (<0.1)0.6 (<0.1)	0.3 (<0.1)0.2 (<0.1)	0.3 (<0.1)0.2 (<0.1)	0.5 (<0.1)0.4 (<0.1)	0.5 (<0.1)0.4 (0.1)
G0-N[H3N3]		0.4(<0.1)0.4(<0.1)	n.d.n.d.	1.1 (0.1)1.1 (0.1)	1.1 (0.1)1.3 (0.1)	1.2 (<0.1)0.9 (<0.1)	1.3 (0.1)0.9 (0.1)	0.7 (<0 .1)0.7 (0.1)	0.2 (<0.1)0.2 (<0.1)	0.2 (<0.1)0.2 (<0.1)	0.2 (<0.1)0.2 (<0.1)	0.8 (0.1)1.0 (<0.1)	0.2 (0.1)0.1 (<0.1)
M5[H5N2]		1.5 (<0.1)1.6 (<0.1)	n.d.n.d.	2.4 (0.1)2.3 (0.1)	1.9 (0.2)1.9 (<0.1)	2.9 (0.1)3.1 (0.1)	1.2 (0.1)1.3 (0.1)	1.9 (<0.1)1.9 (0.1)	0.5 (<0.1)0.5 (<0.1)	1.1 (0.1)1.0 (<0.1)	1.1 (0.1)1.0 (<0.1)	0.9 (<0.1)0.9 (0.1)	1.0 (0.1)0.9 (0.2)
M6[H6N2]		0.1 (<0.1)0.1 (<0.1)	n.d.n.d.	n.d.n.d.	0.4 (0.1)0.3 (0.1)	0.2 (<0.1)0.2 (<0.1)	0.1 (<0.1)0.1 (<0.1)	n.d.n.d.	n.d.n.d.	n.q.n.q.	n.q.n.q.	n.d.n.d.	n.d.n.d.
G1FS[H4N4FS1]		0.2 (<0.1)0.2 (<0.1)	n.d.n.d.	1.3 (0.1)1.4 (0.1)	0.4 (0.1)0.4 (<0 .1)	0.4 (<0.1)0.1 (<0.1)	0.4 (<0.1)0.3 (<0.1)	n.d.n.d.	0.9 (<0.1)0.9 (<0.1)	n.q.n.q.	n.q.n.q.	n.d.n.d.	0.3 (0.1)0.2 (<0.1)
G2S1F[H5N4FS1]		0.7 (<0.1)0.7 (0.1)	n.d.n.d.	1.9 (0.2)1.8 (0.1)	0.6 (0.1)0.6 (0.1)	1.1 (<0 .1)0.4 (<0.1)	0.9 (0.1)0.6 (0.1)	1.4 (0.1)1.5 (0.1)	2.1 (0.1)2.2 (0.1)	n.q.n.q.	n.q.n.q.	n.d.n.d.	0.7 (0.1)0.5 (<0.1)
G2S2F[H5N4FS2]		0.1 (<0.1)0.1(<0.1)	n.d.n.d.	1.0 (0.1)1.0 (0.1)	0.2 (0.1)0.2 (<0.1)	0.2(<0.1)<0.1 (<0.1)	0.3 (<0.1)0.3 (<0.1)	0.4 (0.1)0.5 (0.1)	n.d.n.d.	n.d.n.d.	n.d.n.d.	n.d.n.d.	0.3 (0.1)0.1 (0.1)

The ESI-MS Heavy Chain method allowed only the detection and quantitation of the major galactosylated glycoforms (**Fig. 1** and **Table 2**). ESI-MS after IdeS (FabRICATOR®) allowed the detection of 12 species, of which 11 species could be quantified. The G1 glycostructure [H4N4] could not be separated from the G0F [H3N4F1] glycoform. With the ESI-MS Glycopeptides method, all 14 glycan species could be detected and quantified. With regards to the methods based on LCMS detection of tryptic peptides, the Orbitrap and Synapt G2 MS also facilitated the detection and quantitation of all 14 peaks; however, the fast nano-LCMS of tryptic digests using the Maxis impact™ MS could only detect and quantitate 12 peaks. The same holds true for the PGC-MS analysis of released glycans. The two MALDI-MS analytical methods for glycopeptides (in positive and negative mode) allowed the detection of 13, and the quantitation of 10, glycan species. The MALDI-MS methods based on the detection of released glycans could detect and quantitate 10 peaks (without stabilization of sialic acid) and 13 peaks (with modification of sialic acids).

Three methods allowed the detection and quantitation of 3 sialic acid-containing glycans, namely G1FS [H4N4F1S1], G2S1F [H5N4F1S1] and G2S2F [H5N4F1S2]: 1) ESI-MS after IdeS; 2) ESI-MS Glycopeptides (2 of the 3 methods based on LCMS detection of tryptic peptides); and 3) MALDI-MS Stabilized Glycans. The sialic acid-containing variants could not be detected at all using 2 methods: ESI-MS Heavy Chain and MALDI-MS Glycans. The fast nano-LCMS method for tryptic digests on the Maxis impact™ MS did not detect G1FS, and the PGC-MS method could not detect and quantitate G2S2F.

Concerning the high-mannose glycan species, the M5 [H5N2] was detected and could be quantified by all methods with the exception of ESI-MS Heavy Chain, whereas M6 [H6N2], because of its low abundance, could only be quantified with ESI-MS Glycopeptides and 2 of the trypsin-based LCMS methods.

Mono-antennary structures (with 3 N-acetylglucosamines, such as GOF-N [H3N3F1], G1F-N [H4N3F1] and G0-N [H3N3]) could be detected and quantified with all methods employed except for ESI-MS Heavy Chain. In general, with the exception of ESI-MS of HC after reduction and not considering the sialic acid-containing species, all significant glycan variants could be detected with excellent quantitation performance, and results were comparable to those of the separation-based methods previously reported (**Table 2**).^24^

The total ion chromatogram (TIC) of the PGC-MS method showed some additional minor peaks that could not be unambiguously identified as glycans. However, this might be due to the fact that the method is not run routinely in one of the laboratories that participated in this study, and, with further method development, this may be avoided. The occurrence of these peaks could not be explained, and this could be a potential disadvantage of the method for use in *de novo* glycoanalysis of an unknown sample. The detection of low-abundance glycans was not the focus of this study. However, with some of the mass spectrometry-based methods (LCMS methods, MALDI-MS glycopeptides and MALDI-MS Stabilized Glycans), some glycan species below the limit of quantitation could also be detected, namely H5N5F1 (G2F with bisecting GlcNAc or triantennary), H4N3FS (G1FS-N); the high mannose structures H7N2 (M7), H8N2 (M8) as well as H4N3 (G1-N), H6N3, H6N3F1, H4N3FS (G1FS-N), H5N5F1 (G2F-N). For a better comparison with the separation-based methods, and for simplicity, they were not included in **Table 2**.

### Method performance with regard to accuracy and precision

A summary of the evaluation of the quantitative methods is shown in **Table 2**. As we found in the first part of the study, the reference method HILIC(2-AB) showed excellent precision with low standard deviations, and only minimal differences in average relative abundance were observed between the 2 series (consisting of 6 replicates) analyzed on different days. The MS methods tested in this study also showed low absolute intra-day variation, with values below 1% (with the exception of one analysis series from the fast nano-LCMS with Q-TOF measurements) for all glycan structures.

Importantly, there were only minor differences between the mean results obtained on different days for all methods. However, the inter-day differences in relative intensities of all glycan species were slightly higher than those obtained with the separations methods with a maximum difference of 2.6% (G0F with LCMS with Orbitrap) compared to below 1% for all separation-based methods.^24^ For the major glycan structures, the relative amounts determined by the various MS-based methods were in good agreement with the values obtained with the reference method. For HILIC(2-AB), the G0F species showed an average relative abundance of 35.4%. This value was found to be higher for PGC-MS, MALDI-MS Glycopeptides and MALDI-MS Glycans, ranging from 36.2% to 38.4%, and lower for the other evaluated MS methods, ranging from 29.6% to 34.9%. The G1F species was found with a relative abundance of 43.4% with HILIC(2-AB). This value was found to be higher for ESI-MS Heavy Chain and all MALDI-MS-based methods, ranging from 44.8% to 47.9%, and lower for all other ESI-MS-based systems, ranging from 39.0% to 42.7%. The relative intensity of the doubly galactosylated, fucosylated species G2F was determined to be 9.6% by HILIC(2-AB). The abundances determined with ESI-MS after IdeS were higher with an average amount of 11.7%, while abundances determined by MALDI-MS Glycans and Glycopeptides in positive and negative mode were lower, ranging from 6.7% to 7.3%. All other methods showed G2F levels highly similar to those obtained with the reference HILIC(2-AB) method.

As a parameter for antibody effector function (i.e., ADCC), the sum of afucosylated species (G0+G1+G2) is of biological relevance. For the reference HILIC(2-AB) method, afucosylated species averaged 8.4%. For the ESI-MS Heavy Chain and ESI-MS after IdeS methods, the evaluation was not feasible due to low signal intensities. One LCMS method (nano-LCMS with Q-TOF) was below that level (8.1%). In PGC the relative amount of afucosylated glycan structures was also found to be lower (6.7%). In MALDI-MS Glycopeptides, the afucosylatd glycan levels were 6.0% in positive ion mode and 5.8% in negative ion mode. The other methods showed higher amounts of afucosylated species, namely ESI-MS Glycopeptides (10.5%), the remaining 2 tryptic digest-based LCMS methods (12.0% and 9.7%) and the 2 methods based on MALDI-MS (8.5% and 9.5%). Since small differences in afucosylated glycans might also affect ADCC, the LCMS with tryptic digestion still needs improvement in accuracy for the reliable quantitation of these very important species.

The sum of monoantennary structures (structures lacking an N-acetylglucosamine, i.e., G0F-N, G1F-N and G0-N) was found to average 0.9% for the reference HILIC(2-AB) method. While only low levels were detected with most of the methods (**Table 2**), 3 of the 4 methods relying on ESI-MS for the analysis of glycopeptides detected elevated levels of these glycan variants (ESI-MS Glycopeptides, LCMS with Orbitrap, and LCMS with Q-TOF). The elevated levels may in part be caused by in-source fragmentation of biantennary structures resulting in the loss of one antenna. This loss of an antenna is generally accompanied by a charge reduction, and, accordingly, the resulting fragmentation products show a reduced charge state. These glycan species were found to occur to more than 95% in double-charged form. The LCMS with Q-TOF method showed the most prominent occurrence of these potential in-source decay products (5.6% and 2.7% relative abundance for H3N3F1 and H4N3F1, respectively). Notably, the related nano-LCMS with Q-TOF method (method 7) did not show elevated levels of these glycan species, indicating that in-source decay can efficiently be avoided in ESI-MS analysis of glycopeptides with proper choice of the MS conditions(source and ion optic) and potentials.

Concerning the M5 species, an average relative abundance of 1.5% was detected with HILIC (2-AB). With ESI-MS Heavy Chain analysis, it could not be detected unequivocally and therefore could not be quantified. Similar average values were found with most of the MS-based methods. Higher values were obtained using LCMS with Orbitrap (3%), and lower values were found using PGC-MS (0.5%). The high mannose glycan structure M6 could only be detected and quantified with 4 of the methods (HILIC (2-AB), ESI-MS Glycopeptides, and the LCMS with Orbitrap and Q-TOF), with similar results obtained with all.

The sum of sialylated structures (G1FS, G2S1F and G2SF) was found to be 1.0% with the HILIC (2-AB) reference method. As discussed in the previous section, detection, and hence quantitation, of the sialylated glycan structures was not possible with the ESI-MS Heavy Chain, positive and negative MALDI-MS Glycopeptides and MALDI-MS Glycans methods. Very similar mean amounts to those obtained with HILIC(2-AB) were measured with ESI-MS Glycopeptides (1.2%), LCMS with Orbitrap (1.2%), LCMS with Q-TOF (1.4%), nano-LCMS with Q-TOF (1.9%) and MALDI-MS Stabilized Glycans (1.0%). The values obtained with ESI-MS after IdeS (4.2%) and PGC-MS (3.0%) show a significantly higher bias.

Adjustment of method sensitivity was not within the scope of the study. The limit of detection and the limit of quantitation was only evaluated for the relative amounts for quantification of the ESI-MS Heavy Chain method and was found to be 0.8% and 2.5%, respectively. For the other methods, the sensitivity could only be estimated. For ESI-MS after IdeS, the quantitation limit was also estimated to be around 2.0%. For all other methods, the quantitation limits were estimated to be about 0.1%.

It must be mentioned that the LC-MS methods with tryptic digest can not only detect and quantitate glycan variants, but are also suited to identify and quantitate a large number of other post translational modifications, making these approaches very valuable.

### Analysis time and throughput

The glycopeptide ESI-MS method was developed as a high throughput method.^13^ It is highly automated and limited hands-on time is required. Up to 96 samples can be prepared in parallel. The same holds true for the glycopeptide positive and negative MALDI-MS methods. The other MS-based methods were not specifically developed for high throughput; however, for the ESI-MS Heavy Chain and ESI-MS after IdeS methods, only reduction of heavy chains or digestion with IdeS, respectively, followed by a buffer exchange is needed prior to acquiring MS spectra. As such a high throughput approach for these methods is also feasible. The same holds true for MALDI-MS Glycans since separation prior to analysis is not necessary. However, if quantitation of the sialylated species is also desired, the MALDI-MS Stabilized Glycans method with sialic acid derivatization has to be applied, which requires a more sophisticated and time-consuming sample preparation procedure.

For the other methods (LCMS-based methods of tryptic digest and PGC-MS), the analysis time and throughput are quite similar to the separation-based methods described in the first part of the study (4 hours sample preparation in total, of which about 1.5 hours is hands-on time).^24^

### Required sample amount and purity

As was true for the first part of the study, formulated bulk material, which is of high purity, was used for the investigations, and in fact no interference from contaminants was observed using the reference method. With the MS methods, no labeling procedures were performed and so contaminants from labeling could be excluded. However, if there are contaminants of the same mass as the glycans/glycopeptides, they cannot be differentiated with some of the MS methods where there is no separation before detection. If the mass spectrometer has a very high resolution power (between 60000 and 100000, as in the case of the Orbitrap-MS, it is possible, however, to distinguish molecules that bear the same nominal mass.

Since we did not face sample amount limitations of reference material, the methods were not challenged in terms of sensitivity. The sample amounts (**Table 1**) are in the same range as those used for the separation methods compared in the first part of the study, with one example being DSA-FACE that started with 5 µg of MAb1.

## Discussion

Taken together, with the exception of ESI-MS Heavy Chain, the most prominent glycan species could be detected and quantified with high accuracy and precision using the methods we evaluated. In addition, the results obtained with all separation-based methods (those without mass spectrometric detection) and the mass spectrometric methods were very similar in regard to the robustness and accurate detection and quantitation of low abundance glycoforms.

All MS-based methods may be compromised by in-source decay. Thus, it is not always possible to distinguish between glycan species present in the sample and species produced by in-source fragmentation. This holds true for the bi- and mono-antennary structures. This in-source fragmentation could lead to experimental artifacts; for example, if G0F-N is produced from G0F by fragmentation, G0F will be slightly underestimated. This could partially explain the lower amounts of G0F found for LCMS with Orbitrap and LCMS with Q-TOF compared to the reference method. Accordingly, there are also differences in the relative amounts of G0F-N and G1F-N in the 3 LCMS based methods for glycopeptide analysis, most probably due to low degrees of in-source decay. Consequently, the ionization parameters must be carefully evaluated to avoid fragmentation in LC-ESI-MS of tryptic glycopeptides. However, as shown for the nano-LCMS with Q-TOF data this phenomenon can be avoided. Loss of sialic acids, which represents a common phenomenon in MALDI-MS, could be avoided by derivatization of the carboxyl group. With the MALDI-MS Stabilized Glycans method, it is also possible to distinguish between α2,3 and α2,6 linked sialic acids.^23^ In summary, the fragmentation of sugar moieties observed for MS-based approaches can be limited by an appropriate method development strategy and meaningful system suitability criteria (e.g., for monitoring and controlling in-source decay).

The ESI-MS Heavy Chain method is not suited for a detailed glycosylation characterization of a therapeutic antibody. However, it could be used for a high throughput analysis of the main glycan species. The ESI-MS after IdeS method could not quantitate the afucosylated species G1 and G2. However, in our lab it is still a very robust method and yielded reliable results for most of the glycan species, including the sialylated species. It must be mentioned, however, that the amount of sialylated species does seem to be overestimated compared to the results of the other methods.

The ESI-MS Glycopeptides method is very much suited to high-throughput because it can be relatively easily automated. The same holds true in principle for the other MS methods. The method is also suitable for detecting sialic acid-containing glycans. Together with the DSA-FACE (APTS) system with a DNA sequencer method presented in the first part of our study, ESI-MS Glycopeptides is our preferred method for high throughput analysis. If only minimal amounts of sample are available (for example, for analyzing the glycans after small scale Protein A purification), these 2 methods should be the methods of choice as only about 5 µg of mAb is required.

In contrast, the LCMS methods based on tryptic digestion and subsequent separation prior to the mass spectrometric detection are relatively time-consuming. We compared 3 LCMS methods with mass spectrometers from 3 different vendors in 2 different labs. We observed that the results for different labs and vendors are quite comparable, but it is recommended to use a good and reasonable system suitability sample for the tuning of the mass spectrometer. Data evaluation is more sophisticated and time-consuming for the LCMS methods. The site-specificity of the glycopeptide-based methods may likewise be considered an interesting and valuable distinguishing feature that could be useful, for example in cases where a Fab-glycosylated mAb is to be analyzed. The PGC-MS method needs the highest user expertise because it easily shows artifacts. For unknown reasons, one sialic acid-containing glycan observed with most other methods was not detected with PGC-MS. However, concerning the quantitation of the other glycan peaks, the PGC results are comparable to the other methods tested.

Interestingly, with the MALDI-MS Glycopeptides methods we found almost identical results in positive and negative ion mode, but the method cannot be used for quantitation of sialic acid-containing peptides. The loss of sialic acid can be avoided by using ESI-MS (ESI-MS Glycopeptides method) or by stabilizing the sialic acids prior to MALDI-MS (MALDI-MS Stabilized Glycans method).

With the MALDI-MS Glycans method, the sialic acid-containing glycans also cannot be determined because they are fragmented in the ionization process. However, for the detection and quantitation of all other glycans, the method is very well suited, with the results being very comparable to those of the reference method. The MALDI-MS approach could also be automated due to relatively simple sample preparation and data evaluation steps. The MALDI-MS Stabilized Glycans method has the additional advantage that it is possible to quantify the sialic acid-containing glycans, and to distinguish between α2,3 and α2,6 linked sialic acids. However, the sample preparation is somewhat more laborious. In principle, the MS-based methods potentially can show a bias because the sensitivity is dependent of the ion-transfer, but this can be taken into account by using suited internal standards. Taken together, the MS-based methods are all suited for the detection and quantitation of Fc glycosylation. In principle, they could also be used as release methods. However, the validation of mass spectrometric methods in a GMP environment is a challenge, and, in our hands, the HILIC(2-AB) method was found to be the best-suited release method concerning robustness, accuracy and reproducibility.

## Materials and Methods

MAb1 was produced in the CHO cell line and purified by the Downstream Processing Group at Roche GmbH.

### HILIC(2-AB)

The methodology has been previously described in detail.^24^

### ESI-MS heavy chain

MAb1 samples (250 µg) were diluted to give 200 µl with denaturation buffer (6 M guanidinium-hydrochloride). For reduction, 30 µl of 0.1 M Tris(2-carboxyethyl)phosphine hydrochloride (TCEP; Alfa Aesar, Karlsruhe, Germany) was added and the mixture incubated for 1 h at 37°C. The buffer was exchanged to 250 µl ESI solvent (1% formic acid in 20% ACN) with NAP™-5 columns (Sephadex G-25 DNA Grade; GE Healthcare UK Limited Little Chalfont, Buckinghamshire, UK) using the following procedure. After equilibration of the NAP™-5 columns with 10 ml of ESI buffer, 200 µl of the reduced MAb1 sample was applied. The columns were allowed to elute to completion and then washed with 650 µl of ESI-buffer. 250 µl of ESI-buffer was applied to the columns and the eluates were collected with Eppendorf tubes. All measurements were performed in positive-ion mode with a Waters Synapt® G2 HDMS (Manchester, UK). The *m/z* range was 700–2000. Data acquisition was controlled with the MassLynx™ software (Waters). Raw data were converted into spc-Files (spectra format) and quantified with the GRAMS® software (Thermo Scientific™, Dreieich, Germany). Relative intensities for the signals (peaks) for all charge states for all expected glycovariants of the heavy chain were evaluated and the relative abundances of glycoforms were obtained by normalization to the total glycovariant signal.

### ESI-MS after IdeS (FabRICATOR®, IdeS protease)

Antibody samples (100 µg) were mixed with FabRICATOR® (Genovis, Lund, Sweden) (2 µg in 2 µl Tris HCl buffer, pH 8.0) and diluted with the same buffer to 1 µg/µl Mab1. The sample was incubated at 37°C for 2 h. Samples were buffer exchanged to ESI-buffer (1% formic acid in 20% ACN) with 100 kDa cut-off Vivaspin® centrifugal concentrators. 100 µl of antibody and 400 µl electrospray medium were pipetted into the filters and centrifuged at 10000 rpm for 4 min. 400 µL of ESI-buffer were added twice followed by centrifugation. The samples were diluted to 100 µl in Eppendorf tubes with ESI-buffer.

Samples were analyzed by direct infusion at a flow rate of 7 µl/min into the Synapt® G2 HDMS mass spectrometer (Waters Corp., Manchester, UK) equipped with a standard ESI source. Mass spectra are recorded for at least 3 min with a scan rate of 10 seconds/scan. The *m/z* range was 700–2000. ). Raw data were converted into spc-Files (spectra format) and quantified with the GRAMS® software (Thermo Scientific™, Dreieich, Germany). Relative intensities for the signals (peaks) for all charge states for all expected glycovariants were evaluated and the relative abundances of glycoforms were obtained by normalization to the total glycovariant signal.

### ESI-MS glycopeptides

The method and data evaluation were previously described.^13^ Briefly, MAb1 was digested with trypsin, enriched by HILIC-SPE using Sepharose beads and directly injected in an ESI-MS instrument.

Raw data were converted into spc-Files (spectra format) and quantified with the GRAMS® software (Thermo Scientific™, Dreieich, Germany) using specific ion chromatograms (SICs) for the expected glycopeptides. All detected charge states and isotope peaks of a glycopeptide were summed and the relative abundances of glycoforms were obtained by normalization to the total glycopeptide signal.

### LCMS with Orbitrap

Antibody samples (350 µg) were diluted with denaturation buffer (0.4 M Tris/HCl, 8 M guanidinium pH 8.5) to a final volume of 300 µl. For the reduction, 10 µl of DTT-solution (1 mg/ml) was pipetted to the diluted samples and incubated for 60 min at 50°C. After the incubation, 10 µl of iodoacetic acid (3.3 mg/ml) was added and the solution incubated for 30 min at room temperature in the dark. For the buffer exchange, NAP™-5 columns were equilibrated with 10 ml of digestion buffer (0.1 M Tris/HCl pH 7.0), following which 300 µl of the reduced and carboxymethylated samples were applied and the flow-through discarded. Following a washing step using 350 µl of digestion buffer (eluate discarded), 500 µl of digestion buffer was applied to the columns and the eluate was collected. Following the buffer exchange, 10 µl of a 0.25 mg/ml trypsin solution was added to each sample solution. The samples were incubated in a heating block at 37°C for about 18 h. The digest was separated using an ACQUITY UPLC BEH C18 column (2.1 × 150 mm, 1.7 µm, Waters) with a Waters Acquity UPLC system. The flow rate was 150 µl/min. The gradient was linear from 1% B to 35% B from 0 to 45 min. Solvents A and B were 0.1% formic acid in water and ACN, respectively. All measurements were performed with an Orbitrap Velos mass spectrometer in FTMS mode. Resolution was set to 30000 and the time was 48 min. The detection mass range was set to *m/z* 200–2000. Raw data were acquired by means of the software XCalibur 2.1 provided by Thermo. Raw data were converted into the “spectra format” (SPC), and glycopeptide data analysis was performed as specified above for the Synapt “LCMS with Q-TOF” experiments.

### LCMS with Q-TOF

Samples were prepared as described above for the Orbitrap MS.

Measurements of the tryptic digests were performed in positive-ion mode with a Synapt ESI-quadrupole-TOF-MS instrument (Waters, Manchester, UK). The acquisition time per sample was set to 48 min with a scan duration of 1 s. The detection mass range was set from *m/z* 200–2000.

Data acquisition was controlled by MassLynx software (Waters). Raw data were converted into the “spectra format” (SPC) and quantified with GRAMS software (Thermo Scientific, Waltham, MA) using SICs for the expected glycopeptides. All detected charge states and isotope peaks of a glycopeptide were added and the relative abundances of glycoforms were obtained by normalization to the total glycopeptide signal.

### Nano-LCMS with Q-TOF

Antibody samples (20 µg) were diluted to 20 µl total volumes with 25 mM NH_4_HCO_3_ buffer (pH 8). Two µl SDS in 50 mM NH_4_HCO_3_ buffer (pH 8) was added to achieve a final concentration of 0.02% SDS. The mixture was incubated for 15 min at 60°C. Two µg of trypsin in 18 µl 25 mM NH_4_HCO_3_ buffer (pH 8) was then added and the solution incubated at 37°C overnight. Subsequently, the glycopeptides were purified by HILIC-SPE using cotton wool tips following a procedure similar to a previously published method.^26^ The SPE tips were washed with 3× 20 µl water and conditioned with 3× 20 µl 85% aqueous ACN. Ten µl of the incubation mixtures were diluted with 58 µl ACN. This dilution was loaded onto the SPE tip by aspiration and ejection of 20× 20 µl. Then, the loaded SPE tip was washed 3× with 85% aqueous ACN containing 1% TFA and followed by 3 washes with 85% aqueous ACN lacking TFA. Ultimately, the glycopeptides were eluted in 10 µl water.

Aliquots (5 µl) of the purified tryptic digests were diluted 1:4 in water and 1 µl of the resulting 20 µl sample was subjected to nanoRP-LC–ESI-MS analysis as reported elsewhere.^27^ Briefly, the sample was injected into a 25 µl flow of 0.1% TFA on an Ultimate 3000 LC system (Dionex, Sunnyvale, CA). The glycopeptides were trapped on a C18 trap column (5 mm length × 300 µm I.D.; Acclaim PepMap100; Dionex). The trap column was then switched in line with a 50 mm × 75 µm Ascentis Express C18 nanoLC column with 2.7 µm fused core particles (Supelco, Belfonte, PA, US) to separate the glycopeptides from the residual tryptic peptides, which would interfere with MS detection. The binary gradient was modified from the previously reported method as follows: Linear from 3% to 6% B in 2 min; after 1 min trap column and nanoLC column are switched in line; linear from 6% to 18% from 2 to 4.5 min and to 30% at 5 min; isocratic for 2 min; linear from 30% to 1% from 7 to 8 min and re-equilibration at 1% for 2.9 min. Solvents A and B were 0.1% TFA and 95% ACN, respectively.

NanoESI was achieved with a sheath flow sprayer employing a 2 µl/min sheath flow of 5:2:3 isopropanol:propionic acid:water. A Maxis impact™ quadrupole-TOF (Bruker Daltonics, Bremen, Germany) was used in full spectrum mode at *m/z* 500–2000 from 3 to 8 min for the MS measurements. The areas of all detected charge states (2+ and/or 3+) of a glycopeptide and the first 3 isotope peaks of each charge state were summed and the relative abundances of the glycoforms were obtained by normalization to the total glycopeptide signal.

### PGC-MS

A total of 450 µg of the antibody samples (17.5 µl) were made up to 100 µl with ammonium formate buffer (10 mM, pH 8.6) followed by addition of 5 µl PNGase F (250 U/250 µl water, Roche) at 37°C for ca. Eighteen h. The sample was acidified to pH 3 by addition of 3 µl of 5% formic acid and incubated at room temperature for 30 min to prevent the rearrangement from N-acetylglucosamine to N-acetylmannosamine. Before the reduction, the samples were brought to pH 9 by addition of 3 µl of 5% liquid ammonia. The reduction was performed by addition of 100 µl of 1 M NaBH_4_ and incubation at room temperature for ca. Four h. The desalting and cleanup of the samples was done with HyperSep Hypercarb SPE Columns, Thermo. The columns were washed with 500 µl 80% ACN followed by equilibration with 3× 500 µl pure water. The reduced glycans were then transferred to the columns and centrifuged for ca. Thirty s for loading. After washing 2 times with 500 µl water, the samples were eluted with 100 µl 25% ACN.

Before measurement, the samples were diluted with water 1:5. The glycans were analyzed by PGC UPLC using a Thermo PGC Hypercarb column (2.1 × 100 mm, 5 µm) with a Waters Acquity UPLC system. The detection was performed on a Bruker microTOF-Q mass spectrometer. Aliquots of 10 µl were injected and the flow rate was 150 µl/min. The column temperature was set to 60°C. The gradient was linear from 0% B to 10% B from 0 to 10 minutes; linear from 10% B to 19% B from 10 to 40 min and 19% B to 53% B from 40 to 50 min. Solvents A and B were 0.1% formic acid in water and 1% formic acid in ACN, respectively. The detection range was set to *m/z* 200–2200. Data were acquired by means of the software Compass Hystar provided by Bruker Daltonics. The raw data files (*.d) were converted into the “mxXML” and “mmp” format and quantified with the GRAMS software (Thermo Scientific) by SICs for the expected glycans. All detected charge states and isotope peaks of each glycan were added, and the relative amount of glycoforms were calculated.

### Positive MALDI-MS Glycopeptides

The preparation of the purified glycopeptides was the same as described under “Nano-LCMS of a tryptic digest with quadrupole-time-of-flight MS.” 5 µl of the aqueous samples were spotted on an MTP 384 polished steel MALDI target plate (Bruker), dried and overlaid with 2 µl of a 5 mg/ml solution of 4-chloro-α-cyanocinnamic acid in 70% aqueous ACN.^19^ Spectra were collected from *m/z* 1000–4000 in reflectron positive ion mode on an UltraFlextreme MALDI-TOF-MS (Bruker). Acquisition proceeded at 2000 Hz for 5000 shots using a random-walk algorithm to limit the shots to 50 per raster spot. All detected charge states and isotope peaks of a glycopeptide species were added and the relative amount of glycoforms were calculated with in-house developed software.^23^

### Negative MALDI-MS Glycopeptides

The negative ion mode measurements were taken from the same sample and on the same instrument with the following differences: Spectra were acquired over the *m/z* range 1000 to 3800 in reflectron negative ion mode, summing 20,000 laser shots instead of 5,000. Data evaluation was performed as in the positive mode.

### MALDI-MS Glycans

#### PNGase F digest of the antibody

The 10 kDa molecular weight ultrafiltration cups (VIVASPIN 500, Sartorius, Göttingen, Germany) were washed twice with 500 µl distilled water and centrifuged for 5 min at 5,000 g. Then, 75 µg antibody was applied to each ultrafiltration cup. Next, the cups were filled with distilled water to a volume of 500 µl and centrifuged again for 5 min at 5,000g. For buffer exchange, the samples were washed threefold with 450 µl Baker-H_2_O with centrifugation for 5 min at 5,000 g. The concentrated samples of around 50 µl were then transferred to a 1 ml reaction vial. One µl of 5 U PNGase F (Roche, Mannheim, Germany) was added to each sample and incubated for 16 h at 37°C. In the final step, 4 µl of 1.5 M acetic acid was added to each sample, followed by incubation for 3 h at 25°C.

#### MALDI-TOF MS of released glycans

The sample solution (0.5 µl) was mixed with an equal volume of DHB matrix (10 mg/ml 2,5-dihydroxybenzoic acid in 1 ml 10 mM sodium chloride). 1µl of each mixture was spotted on a MALDI target and dried under vacuum. The mass spectra were obtained on a TOF/TOF™ 5800 MALDI-TOF System (AB SCIEX, Framingham) operated in the positive ion reflector mode. For the measurements, laser intensity and delay times were carefully adjusted to prevent glycan fragmentation. All glycans of interest were identified manually by searching for their singly charged *m/z*-values within the experimental mass spectrum. Relative quantification of detected glycan structures was performed by direct comparison of the area values provided by the TOF/TOF™ Series Explorer™ Software (AB SCIEX, Framingham). All detected charge states and isotope peaks of a glycan were added and the relative amount of glycoforms were calculated.

### MALDI-MS Stabilized Glycans

Aliquots of 2 µl sample (50 µg mAb) were mixed with 3 µL PBS and 10 µl 2% SDS for 10 min on a shaker. Afterwards, the mixture was incubated at 60°C for 10 min. A master mix of 100 µl 4% NP-40, 100 µl 5x PBS and 10 µl PNGase F (10 U) was prepared. After cooling to RT, 10 µl of the 0.5 U PNGase F was added to each sample. The glycans were released by overnight incubation at 37°C. Two µL of the released glycans were ethyl esterified^23^ by incubation for 1 h at 37°C after addition of 20 µl of an ethanol solution containing 250 µM each of 1-ethyl-3-(3-dimethylaminopropyl)carbodiimide hydrochloride (Fluorochem, Hadfield, UK) and hydroxybenzotriazole monohydrate (Sigma-Aldrich, Steinheim, Germany). After addition of 20 µl ACN the samples were purified by HILIC-SPE as described in “Nano-LCMS with Q-TOF.”

Aliquots (1 µl) of the aqueous sample was spotted on an MTP AnchorChip 800/384 MALDI target plate (Bruker), dried and overlaid with 1 µl of a 5 mg/ml solution of 2,5-dihydroxybenzoic acid in 50% aqueous ACN containing 1 mM sodium hydroxide. The spots were recrystallized with 0.2 µl of ethanol in order to achieve uniform crystals. Spectra were collected from *m/z* 1000 to 5000 in reflectron positive ion mode on an UltraFlextreme MALDI-TOF-MS (Bruker). Acquisition proceeded at 2,000 Hz for 20,000 shots using a random-walk algorithm to limit the shots to 50 per raster spot. All detected charge states and isotope peaks of a glycan were added, and the relative amount of glycoforms were calculated by an in-house software.^23^
