# Supplementary material for: Comparison of methods for the analysis of therapeutic immunoglobulin G Fc-glycosylation profiles—Part 2: Mass spectrometric methods
Source: MAbs. 2015 May 21;7(4):732–42. doi: 10.1080/19420862.2015.1045173 (PMC4622708; doi:10.1080/19420862.2015.1045173)
Supplement: Supplemental_Material.zip [file kmab-07-04-1045173-s001.zip › Supplemental Material.docx]

**Supplementary Files: Figure Legends**

Figure 1. LCMS with Orbitrap. (A) TIC; (B) MS spectrum of 5.6 – 8.3 min

Figure 2. Nano-LCMS with Orbitrap. MS spectrum of 1.9 – 2.6 min (TIC not available)
